# Supplementary material for: Heavy-element damage seeding in proteins under XFEL illumination
Source: J Synchrotron Radiat. 2025 Aug 27;32(Pt 5):1124–42. doi: 10.1107/S1600577525005934 (PMC12416421; doi:10.1107/S1600577525005934)
Supplement: Supplementary file 1 [file s-32-01124-sup1.pdf]

JOURNAL OF SYNCHROTRON RADIATION

Volume 32 (2025)

Supporting information for article:

Heavy-element damage seeding in proteins under XFEL illumination

Spencer K. Passmore, Alaric L. Sanders, Andrew V. Martin and Harry M. Quiney

1. Supplementary Figures

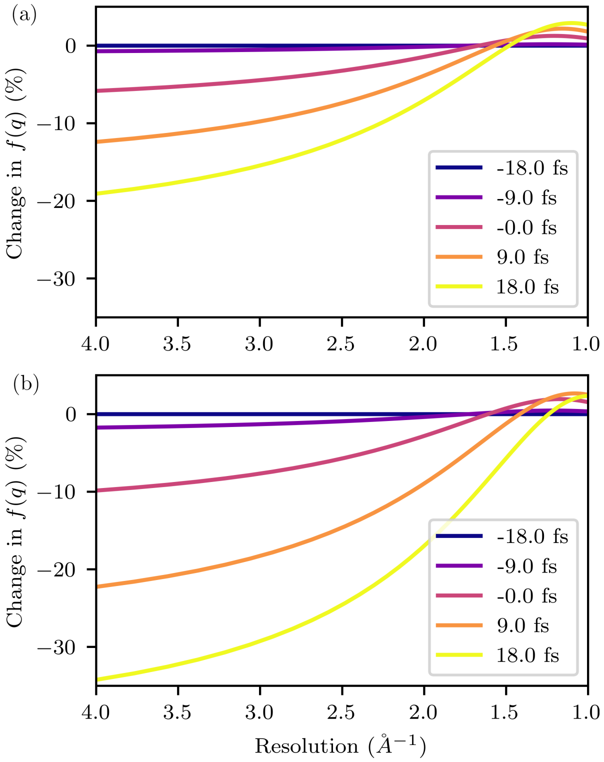

Fig. S1. Evolution of the scattering amplitude of carbon atoms in the lysozyme.Gd protein, under the illumination conditions of Sec. 3.1. (a) Simulation where only light atom ionization is modeled. (b) Simulation where all ionization is modeled. Traces correspond to the 'average' carbon atom in the protein at the denoted times, defined as a carbon atom with the average orbital occupancies of all carbon atoms. The atomic form factor $f(q)$, where q is the momentum transfer, is defined as the Fourier transform of the atom's electron density. The vertical axis gives the change in $f(q)$ for the average carbon atom relative to the form factor of a carbon atom in its neutral ground state. The horizontal axis gives the resolution $(2\pi/q)$ that corresponds to the scattering angle.

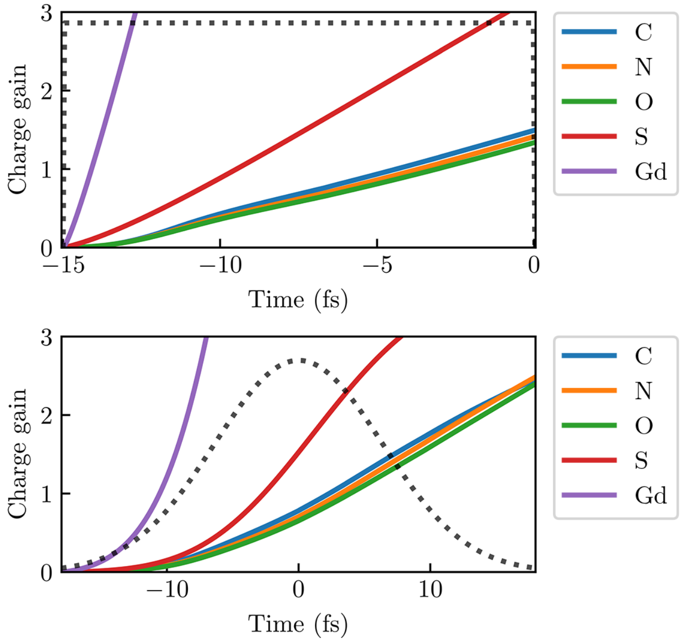

Fig. S2. Effect of the choice of pulse profile used in simulating the dynamics for the lysozyme.Gd crystal with 10% NaCl solvent considered in Sec. 3. Plots show the evolution of the average charges for elements present in the protein under the Gaussian and square pulse profile idealizations for a 15 fs FWHM pulse, as represented by the dotted lines. Both pulses have a fluence of 1.75×10^{12} 7.112 keV ph·µm^{-2}. By $t = 0$, the target is in a more ionized state under the Gaussian pulse.

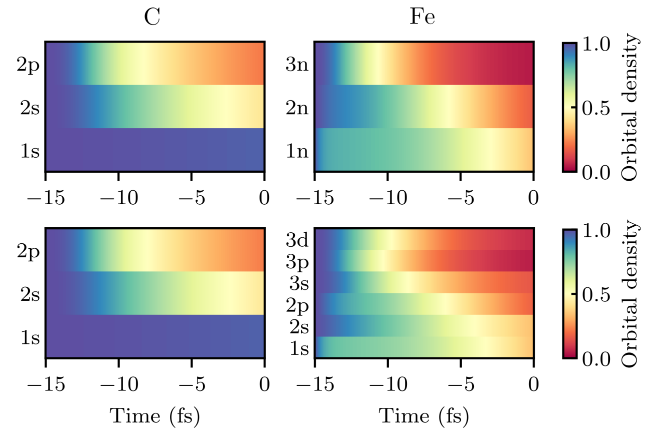

Fig. S3. Occupancy of C and Fe within the Fe-doped protein, with (top) and without (bottom) the single-shell approximation that was applied in this study to atoms heavier than Fe. The pulse was modeled with a 15 fs square temporal profile, and a fluence of 10^{13} 10 keV ph·µm^{-2}. TBR was disabled in these simulations. The single-shell approximation has a negligible impact on the ionization of C.

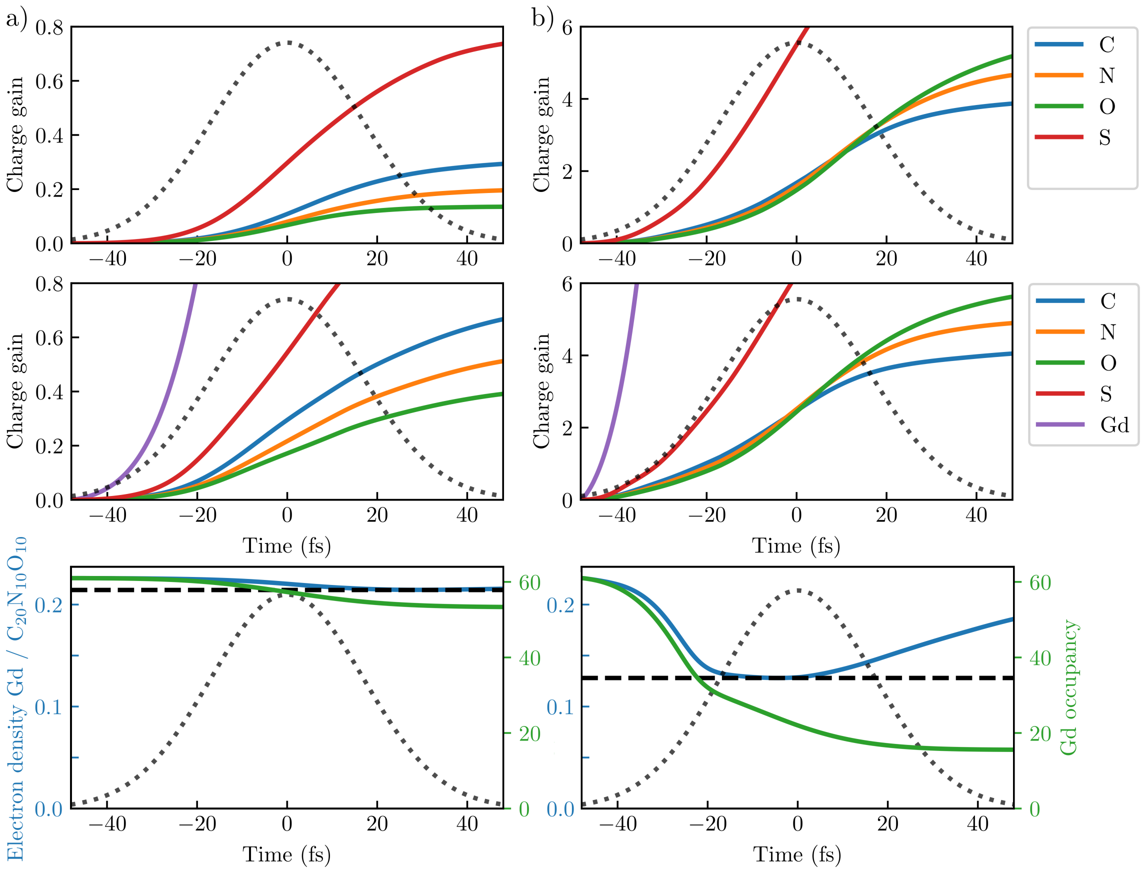

Fig. S4. Effect of Gd ions on the ionization of lysozyme.Gd in the (a) low and (b) high fluence experiments performed by Galli *et al.* [*IUCrJ* (2015) **2**, 627–634]. Pulses were simulated with 40 fs FWHM Gaussian profiles, and (a) 1.3×10^{11} 8.48 keV ph·μm^{-2} or (b) 7.8×10^{12} 8.48 keV ph·μm^{-2} fluence. The photon energy is above the L-edge of Gd (modeled as 7.42 keV). Plots in the top row correspond to lysozyme in water as solvent, and plots in the middle row correspond to the true composition: lysozyme.Gd in 0.1 M Gd 8% NaCl solvent. The presence of Na^+, Cl^-, and Gd^{3+} ions (middle row) increases the ionization of the light atoms. Plots in the bottom row show the evolution of the Gd occupancy and EDR. The horizontal dashed line shows the EDR and Gd occupancy based on the ionization theoretically predicted for each element in the original study, where the quantities are assumed to be commensurate. Note that Gd occupancy is proportional to EDR neglecting light atom damage. The EDR and Gd occupancy diverge significantly during the pulse due to the substantial light-atom ionization.

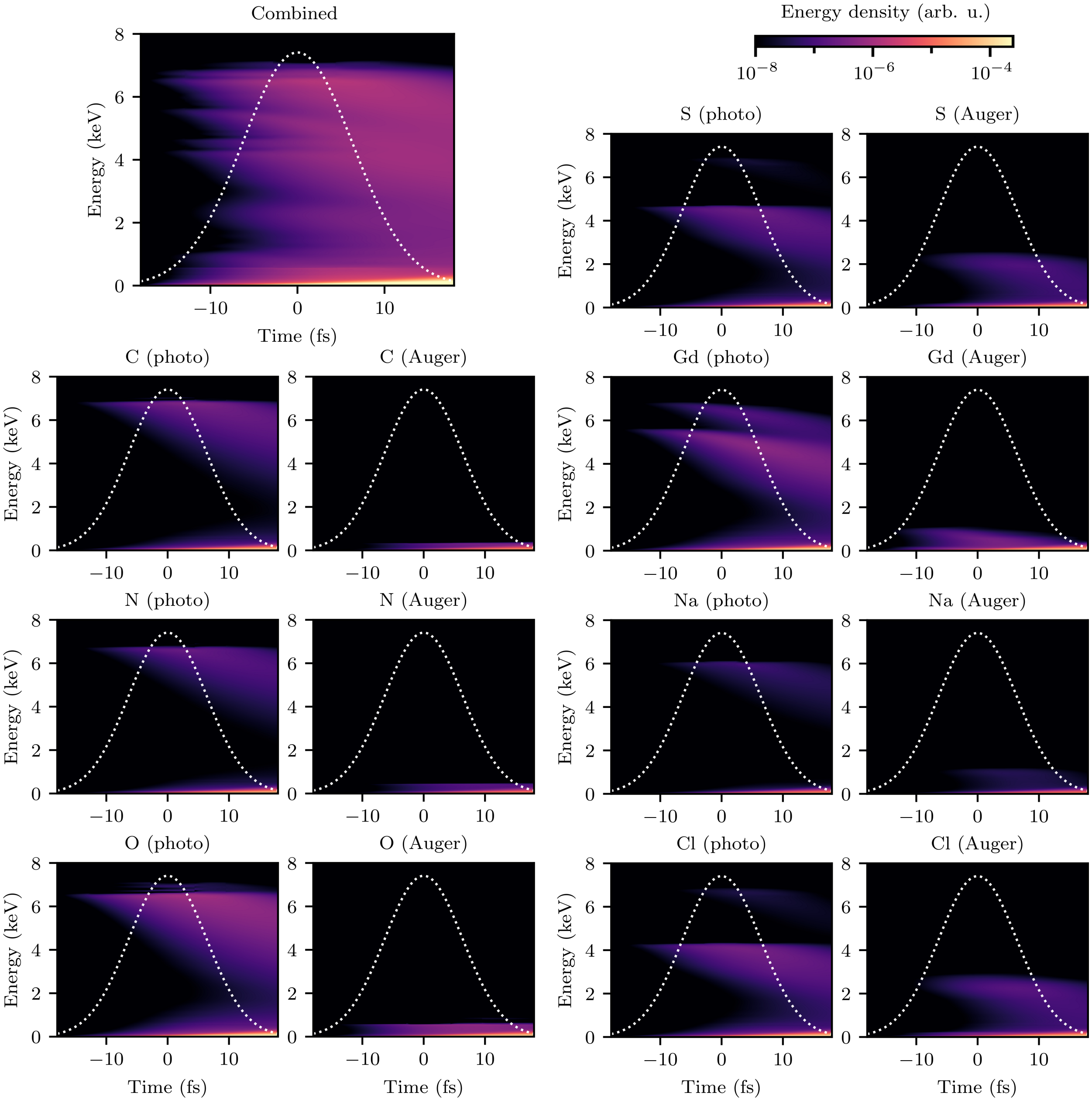

Fig. S5. Contributions of each element to the free-electron cascades in the lysozyme.Gd crystal under the illumination conditions of Sec. 3.1. Each plot shows the energy density of electrons freed from EII cascades seeded by Auger electrons or photoelectrons from an element, as denoted above the plot. Secondary ionization of heavy elements is ignored. The continuums include the contribution of the primary electrons. The ‘combined’ plot corresponds to the complete free-electron continuum.
